# Clonality and the Phenotype–Genotype Correlation of Antimicrobial Resistance in *Acinetobacter baumannii* Isolates: A Multicenter Study of Clinical Isolates from Romania

**DOI:** 10.3390/microorganisms13010176

**Published:** 2025-01-16

**Authors:** Adrian-Gabriel Pană, Pavel Șchiopu, Dan Alexandru Țoc, Vlad Sever Neculicioiu, Anca Butiuc-Keul, Anca Farkas, Matei-Ștefan Dobrescu, Mirela Flonta, Carmen Costache, Izabella Éva Szász, Lia-Monica Junie

**Affiliations:** 1 Department of Microbiology, Iuliu Hatieganu University of Medicine and Pharmacy, 4–6 Louis Pasteur Street, 400012 Cluj-Napoca, Romania; toc.dan.alexandru@elearn.umfcluj.ro (D.A.Ț.); neculicioiu.vlad.sever@elearn.umfcluj.ro (V.S.N.); carmen.costache@yahoo.com (C.C.); monicajunie@yahoo.com (L.-M.J.); 2 Faculty of Medicine, Iuliu Hatieganu University of Medicine and Pharmacy, 400012 Cluj-Napoca, Romania; 3 Doctoral School of Integrative Biology, Babeș-Bolyai University, 1 M. Kogalniceanu Street, 400084 Cluj-Napoca, Romania; anca.keul@ubbcluj.ro (A.B.-K.); ancuta.farkas@ubbcluj.ro (A.F.); matei.dobrescu@ubbcluj.ro (M.-Ș.D.); 4 Department of Molecular Biology and Biotechnology, Faculty of Biology and Geology, Babeș-Bolyai University, 1 M. Kogalniceanu Street, 400084 Cluj-Napoca, Romania; 5 Centre for Systems Biology, Biodiversity and Bioresources, Babes-Bolyai University, 5–7 Clinicilor Street, 400006 Cluj-Napoca, Romania; 6 Infectious Disease Clinical Hospital, 23 Iuliu Moldovan Street, 400003 Cluj-Napoca, Romania; m.flonta@yahoo.com; 7 Cluj-Napoca Emergency Clinical County Hospital, 3–5 Clinicilor Street, 400347 Cluj-Napoca, Romania; 8 Târgu-Mureș Emergency Clinical County Hospital, 50 Gheorghe Marinescu Street, 540136 Târgu-Mureș, Romania; szasz.iza@gmail.com

**Keywords:** antibiotic resistance genes, carbapenem-resistant *Acinetobacter baumannii*, clonality, ESKAPE, Europe, extensively drug resistant, polymerase chain reaction, resistome

## Abstract

Antibiotic resistance is on the WHO’s top 10 list of global public health threats due to its rapid emergence and spread but also because of the high morbidity and mortality associated with it. Amongst the main species driving this phenomenon is *A. baumannii*, a member of the ESKAPE group of medical assistance-associated infections causing species famous for its extensively drug-resistant phenotypes. Our findings note a 91.52% frequency of extensively drug-resistant carbapenem-resistant *A. baumannii* (XDR CRAB) phenotype amongst clinical isolates from multiple hospitals in two major cities from northwestern and central Romania, harboring multiple antibiotic resistance genes such as bla*_OXA-23-like_* in 108 (91.5%) isolates, bla*_OXA-24/40-like_* in 88 (74.6%) isolates, bla*_NDM_* in 29 (25%) isolates, *ArmA* in 75 (63.6%) isolates, and *ant(3″)-I* in 69 (58.5%) isolates and *sul1* in 113 (95.76%) isolates. The isolates, although nearly identical in phenotype, displayed different genotypical profiles, with varying degrees of similarity across hospitals and cities, raising the possibility of both local outbreaks of a single clone and widespread dissemination of resistant isolates.

## 1. Introduction

Antimicrobial resistance is rapidly emerging as one of the most critical public health challenges, approaching almost pandemic-level concerns. *Acinetobacter baumannii*, a Gram-negative, non-fermentative bacillus of the *Moraxellaceae* family, is among the species that most prominently exemplify this issue. In 2018, the World Health Organization (WHO) included *A. baumannii* in the ESKAPE group (*Enterococcus faecium*, *Staphylococcus aureus*, *Klebsiella pneumoniae*, *A. baumannii*, *Pseudomonas aeruginosa*, *Enterobacter* spp.), a collection of highly virulent bacterial pathogens. Furthermore, *A. baumannii* was designated as a top priority in research for antibiotic resistance patterns and novel drug development [1].

The medical importance of *A. baumannii* is determined by both its remarkable ability to survive in hospital environments and its implication in severe healthcare-associated infections with high mortality rates [2]. Multiple mechanisms may explain the environmental fitness and high virulence of *A. baumannii*: the ability to form biofilms on both epithelia and abiotic surfaces, metabolism modulatory capacity in adverse environments, and extensive drug resistance to most classes of antibiotics and antiseptics [3,4,5,6,7]. While some of its resistance is intrinsic (resistance to macrolides, fosfomycin, trimethoprim, penicillin, and cephalosporins), the concern mainly lies with acquired resistance, with three defined phenotypes.

The first phenotype of concern is multi-drug-resistant or MDR *A. baumannii* (resistant to at least one antibiotic from two separate classes). The second extensively drug-resistant carbapenem-resistant *A. baumannii* phenotype is XDR CRAB. Extensive drug resistance is defined as nonsusceptibility to at least one antibiotic from all but two or fewer antibiotic classes. This phenotype has been shown to be the most frequently encountered in clinical settings all across Eastern Europe ever since 2019 [8,9,10,11,12]. CRAB encompasses acquired resistance to carbapenems, aminoglycosides, quinolones, and sulfonamides. In some cases, CRAB can evolve into a third phenotype: pan-drug-resistant (PDR) isolate by acquiring resistance to colistin, a last-resort antibiotic frequently used in CRAB infections [13,14,15,16].

Antibiotic resistance mechanisms can be classified into three categories. Resistance may be attained by limiting access to the target through decreased membrane permeability or enhanced antibiotic efflux [17,18,19], by shielding the target through post-translational modifications or genetic mutations [18,20,21], or by directly inactivating the antibiotic molecule through structural alteration or complete hydrolysis [18,19,22,23,24,25]. The presence of these mechanisms in *A. baumannii* may be explained through its remarkable genetic flexibility. This adaptation allows for quick genetic changes and rearrangements as well as the acquisition of foreign determinants carried by mobile genetic elements (such as plasmids, transposons, integrons, and insertion sequences) [26,27]. By incorporating these elements, containing various types of antibiotic resistance genes (ARGs), *A. baumannii* gains the ability to protect itself from antibiotics from other bacterial species populating the hospital setting, a process known as HGT (horizontal gene transfer) [28].

While most studies that examine XDR CRAB isolates from clinical isolates seem to focus mostly on identifying carbapenemase-producing ARGs, it is equally important to assess the presence of ARGs associated with other classes of antibiotics that are monitored and utilized. The aim of this paper was to provide an extended description of the *A. baumannii* ARG resistome through phenotype–genotype correlation and to highlight the clonality relations between the isolates isolated from multiple hospital units across Romania, a country severely affected by CRAB and with scarce data regarding its isolation and its antimicrobial resistance patterns [10].

## 2. Materials and Methods

### 2.1. Study Design and Setting

A total of 118 clinical isolates of *A. baumannii* from confirmed infections have been collected between January 2023 and May 2024 from three major university hospitals (Cluj-Napoca Emergency County Hospital, Cluj-Napoca Infectious Diseases Clinical Hospital, and Târgu-Mureș Emergency County Hospital) in the largest two cities (Cluj-Napoca, Târgu-Mureș) in the northwestern and central development regions of Romania. For each, clinical isolate data were collected regarding gender, age, hospitalization period, the type of infection caused by *A. baumannii*, and the outcome of the patient (cured, improved, stationary, aggravated, and deceased). No personal or identifiable data were extracted regarding the patients. Furthermore, all patients signed informed consent forms upon admission in all three teaching hospitals. The distinction between colonization and infection was made based on clinical assessment, imaging results, and laboratory tests in accordance with national and international guidelines.

### 2.2. Isolate Identification and Antibiotic Susceptibility Testing

Identification of the isolates was achieved using matrix-assisted laser desorbtion ionization-time of flight mass spectrometry (MALDI-TOF MS) from Bruker (Billerica, MA, USA) with Bruker^®^ flexControl and MBT Compass software (version 4.1.80) and VITEK 2 Compact with VITEK 2^®^ GN identification cards (bioMérieux, Inc., Marcy l’Etoile, France). Antimicrobial resistance was determined using automated MIC determination techniques with VITEK 2^®^ AST cards (AST-N222, N376, N397—bioMérieux, Inc., Marcy l’Etoile, France) and Bruker^®^ Micronaut IVD System 96-well titer plates. Interpretation of the obtained resistance patterns was performed according to the v.14.0 EUCAST 2024 guidelines for the following antibiotics: meropenem, imipenem, amikacin, gentamicin, tobramycin, ciprofloxacin, trimethoprim-sulfamethoxazole, and colistin [29].

### 2.3. Molecular Characterization of Antibiotic Resistance and Clonality

Isolates were tested for 22 ARGs, *mexA* and *mexB* genes encoding the MexAB-OprM efflux pump, integrase genes for class I and II integrons, and insertion sequence IS*Aba1*. For the insertion sequence, its presence upstream of *bla_OXA-23_* and *bla_OXA-51_* was also tested using the forward primer from IS*Aba1* and reverse primer from each specific gene.

Clonality was studied using the Enterobacterial Intergenic Consensus-PCR (ERIC-PCR) molecular typing method as previously described [30]. The list of the tested ARGs, integrons, and ERIC-PCR specific primers (Eurogentec, Liège, Belgium) and their respective amplicon size are found in Table 1, Table 2 and Table 3.

Isolates were stored at −20 °C in a mixture of peptone water and Noble agar and subcultured on Columbia agar with 5% defibrinated sheep blood for genotypic analysis. A 0.5 McFarland bacterial suspension was prepared from each subculture, using distilled water as the suspension medium to facilitate cell wall lysis through hypoosmotic stress. This suspension was used as template for the PCR. An amount of 12.5 μL of DreamTaq Green PCR master mix (2×) (Thermo Fisher Scientific, Waltham, MA, USA), 10.25 μL of nuclease-free water (Lonza, Basel, Switzerland), 25 pmol of each primer (Eurogentec, Belgium), and 2 μL of bacterial suspension were mixed, yielding a 25 μL total reaction volume.

The general PCR program was 5 min at 94 °C initial denaturation stage followed by 35 cycles of 30 s at 94 °C denaturation, 45 s annealing with temperatures varying according to each primer pair (annealing temperatures listed in Table 1, Table 2 and Table 3), and 30 s at 72 °C elongation, with a final elongation of 8 min at 72 °C. For ERIC-PCR, the program had to be modified for the simultaneous accurate detection of multiple amplicons: 5 min at 94 °C initial denaturation stage followed by 5 cycles of 5 min at 94 °C denaturation, 5 min at 38 °C annealing, and 5 min at 72 °C elongation, followed by 30 cycles of 1 min at 94 °C denaturation, 1 min at 48 °C annealing, and 5 min at 72 °C elongation, with a final elongation of 10 min at 72 °C. Negative control was made using only primers, master mix, and nuclease-free water. For the positive control, a bacterial suspension of *A. baumannii* ATCC 19606 was used as template. TProfessional Trio (Analytik Jena, Jena, Germany) and Mastercycler Nexus (Eppendorf AG, Hamburg, Germany) thermocyclers were used for the PCR reaction. Agarose gel electrophoresis was used to separate the amplicons, using 1.5% *w*/*v* agarose (Cleaver Scientific, Rugby, UK) in 1 × TAE buffer (Lonza, Basel, Switzerland) stained with 0.5 μg/mL ethidium bromide (Thermo Fisher Scientific, Waltham, MA, USA) for the preparation of the gel. First well of each row was loaded with 10 μL of 100 bp GeneRuler DNA ladder (Thermo Fisher Scientific, Waltham, MA, USA). The gel was visualized under UV light, and data capture was conducted with the BDA Digital Compact System and BioDocAnalyze Software (version 2.64.11.20, Analytik Jena, Jena, Germany).

### 2.4. Data Analysis

The presence of ARGs amplicons and ERIC-PCR profiles were indicated by the absence (marked 0) or presence (marked 1) of bands in the expected position according to their molecular weight in comparison with the DNA ladder. Data analysis and dendrogram assembly for the ERIC-PCR patterns was conducted with DARwin (Dissimilarity Analysis and Representation for Windows) from Cirad (version 6, Montpellier, France) using a Dice coefficient for the calculus of dissimilarity and an UPGMA (unweighted pair group method with arithmetic mean) for hierarchical clustering for the dendrogram.

R (version 4.4.1, The R Foundation, Vienna, Austria), RStudio (version 2024.09.0+375, Posit Software, Boston, MA, USA), and IBM SPSS Statistics (version 27.0.1.0, Armonk, NY, USA) were used to conduct statistical tests. We used the Chi-square test (two-sided) and Fisher’s exact test (two-sided) on a case-by-case basis to assess the association between categorical variables, taking into account the sample size and distribution of data. Specifically, the Chi-square test was applied when the sample size was large, and the expected frequency in each cell of the contingency table met or exceeded the threshold of 5, ensuring the validity of the test assumptions. Fisher’s exact test was employed as an alternative to the Chi-square test in scenarios where the sample size was small or when the expected frequency in one or more cells of the contingency table fell below 5. Statistical significance was considered at *p* < 0.05.

## 3. Results

### 3.1. General Data

**Figure 1:**
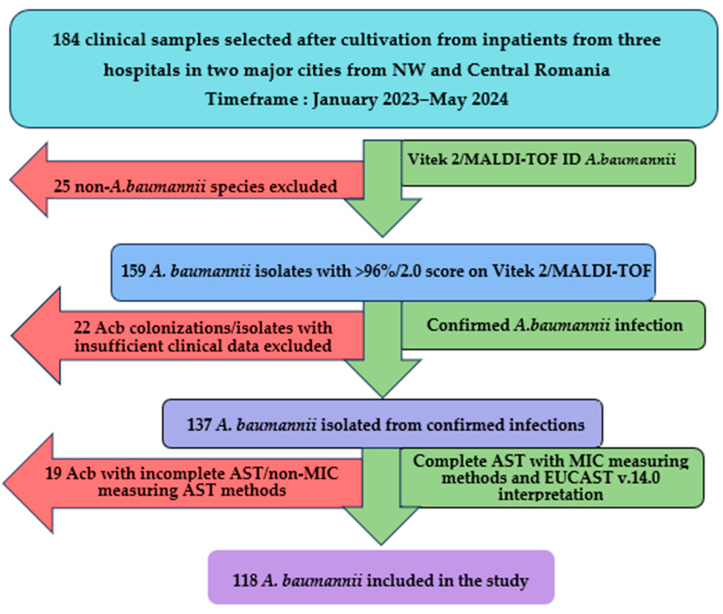
*A. baumannii* isolates inclusion flowchart. Acb—*A. baumannii,* NW—northwestern, AST—antibiotic sensibility testing, MIC—minimum inhibitory concentration.

Between January 2023 and May 2024, a total of 184 clinical samples were analyzed following the culture of Gram-negative, lactose-negative colonies in aerobic conditions at 37 °C for at least 24 h incubation on multiple selective and differential culture media (MacConkey agar, CLED, URISelect by BioRad, Hercules, CA, USA). Following the exclusion of 66 isolates, a total of 118 isolates (64.13%) of *A. baumannii* were included in the study and validated for genotypical and molecular typing analysis, as shown in Figure 1.

Out of the 118 *A. baumannii* isolates included in the study, the majority were isolated from respiratory tract infections (*n* = 54; 45.76%) and sepsis (*n* = 32; 27.11%) of male patients (*n* = 70; 59.32%) between 61 and 75 years (*n* = 45; 38.13%) or older (*n* = 37; 31.35%). Most of the infections were reported in the intensive care unit (ICU) (*n* = 50; 42.37%). An exceedingly high mortality rate was observed (*n* = 64/118; 54.23%), with 40 out of 118 patients (33.89%) being declared improved or cured. Antibiotic therapy was mostly performed with Colistin (*n* = 57, 48.3%) or antibiotic combinations with Colistin (Colistin + Meropenem and Colistin + Tigecycline being the most frequent options used in 21 (17.79%) and 8 cases (6.77%), respectively). More information regarding clinical data about the origin of the isolates is found in Table 4.

### 3.2. Resistance Phenotypes

Out of the 118 included isolates, the XDR CRAB phenotype was the most frequently encountered (*n* = 108; 91.52%). The remaining 10 isolates were classified as susceptible (*n* = 5; 4.23%), MDR (*n* = 1; 0.84%), or PDR (*n* = 4; 3.38%). All XDR isolates showed resistance to both imipenem and meropenem, ciprofloxacin, and trimethoprim-sulfamethoxazole and maintained sensitivity to colistin. Resistance to all three tested aminoglycosides was found in 96 out of 108 XDR isolates (88.88%), with the other 12 isolates (11.11%) being sensitive to one or two of the three aminoglycosides. Three of the PDR isolates were isolated from Târgu-Mureș Emergency County Hospital and the other one from Cluj-Napoca Emergency County Hospital. Three of the isolates were isolated from patients with respiratory tract infections and the other one from a postoperative wound infection. No statistical significance was observed between the resistance phenotype and the hospitals, wards, or type of infection the isolates were isolated from (*p* > 0.05).

### 3.3. Resistance Genotypes

**Figure 2:**
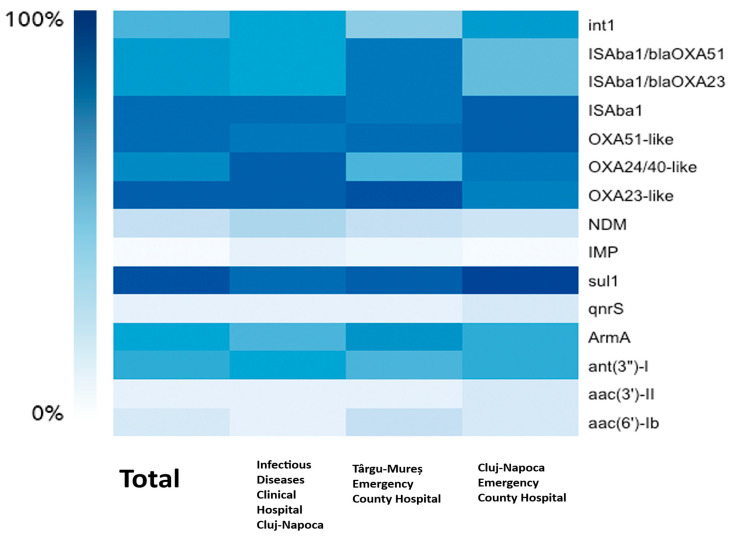
The correlation between the hospitals and the frequency of the ARGs.

For resistance to beta-lactams, we have detected genes encoding Ambler class B MBL (metallo-beta-lactamase) and class D (oxacillinase) beta-lactamases. The analysis encompassed 118 *A. baumannii* isolates confirmed by MALDI-TOF or Vitek 2 analysis, with accuracy scores > 2.0 for MALDI-TOF and >96% identification probability (excellent identification) for Vitek. However, only 105 isolates (89%) were positive for the chromosomally encoded *bla_OXA-51-like_* gene. The *bla_OXA-23-like_* gene was found in 108 (91.5%) isolates and *bla_OXA-24/40-like_* in 88 (74.6%) isolates. No isolate has been found to harbor the *bla_OXA-143-like_* gene. For the metallo-beta-lactamase-encoding genes *bla_NDM_* was found in 29 (25%) isolates and *bla_IMP_* in only 1 isolate (0.84%) from Cluj-Napoca Emergency County Hospital. *bla_VIM_, bla_SIM_*, and *bla_SPM_* were not found in any of the 118 isolates. Insertion sequence *Aba1* (IS*Aba1*) was found in 104 (88%) isolates, while its placement upstream of bla*_OXA-23-like_* and *bla_OXA-51-like_* for the upregulation of the aforementioned gene’s expression was detected in 80 (68%) isolates. Aminoglycoside resistance was tested by searching for various classes of AMEs (aminoglycoside-modifying enzymes) and 16S rRNA methyltransferase. *ArmA* (75; 63.6%) and *ant(3”)-I* (69; 58.5%) were the most common genes found, and *aac(6′)-Ib* and *aac(3′)-II* were less prevalent, occurring in 15.3% and 6.8% of isolates, respectively. *aph(3′)-IIb* was not found in any isolate. For quinolone resistance, Qnr protein-encoding genes were screened for, with only 9 isolates (7.6%) exhibiting *qnrS* without statistical significance (*p* = 1) and none exhibiting *qnrA* and *qnrB*. With a prevalence of 113 out of 118 isolates (95.8%), the sulfonamide resistance gene sul1 is the most frequent ARG found in our study, being statistically significant as well (*p* = 0.02). No PDR isolate tested positive for *MexA* and *MexB* or *mcr-1* genes. Last but not least, 64 isolates (54.2%) had the class 1 integron gene *int1*, a crucial indicator for gene acquisition and horizontal gene transfer. Details about the phenotype–genotype correlations and statistical significance for beta-lactams and aminoglycosides are displayed in Table 5 and Table 6. A heatmap of the frequency of the ARGs for each hospital can be found in Figure 2.

### 3.4. Clonality

**Figure 3:**
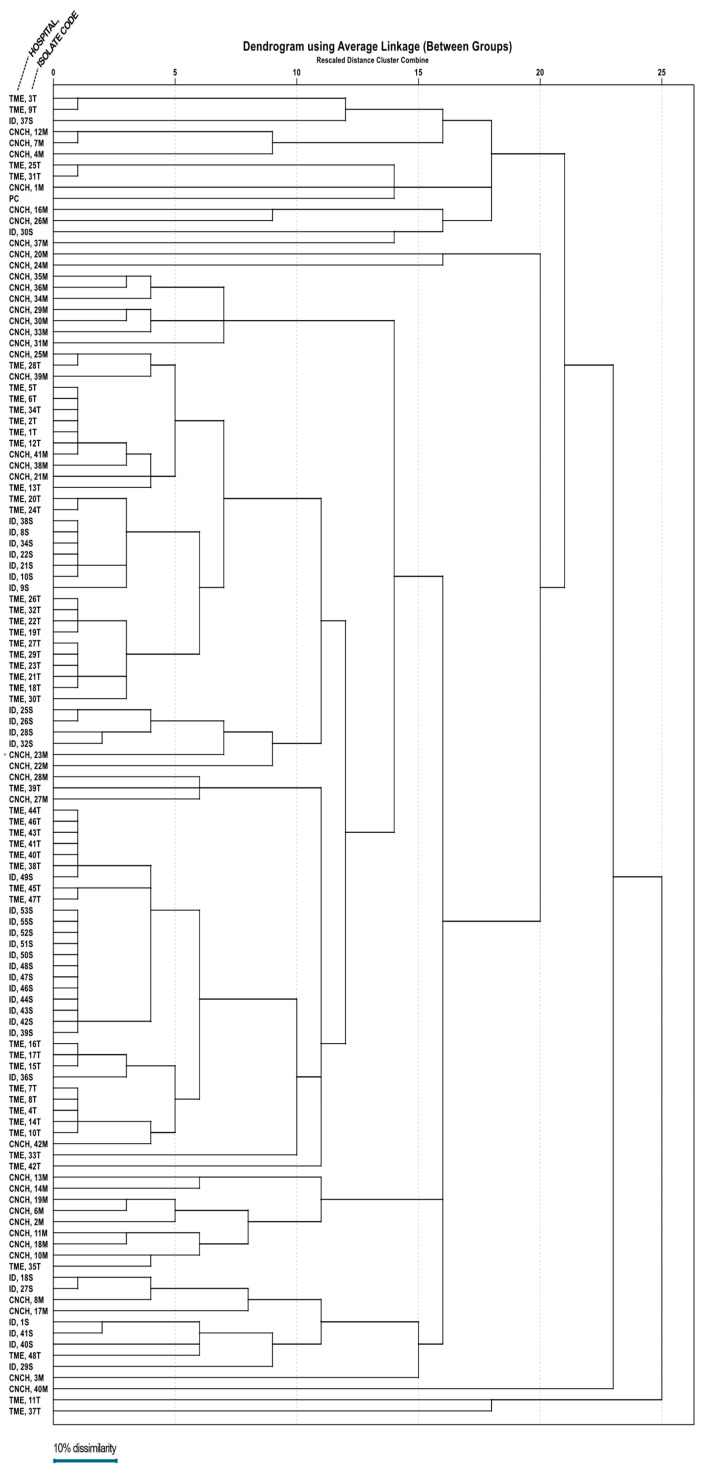
The dendrogram representing clonality relations of 118 *A. baumannii* strains isolated from the three major hospitals in northwestern and central Romania.

**Figure 4:**
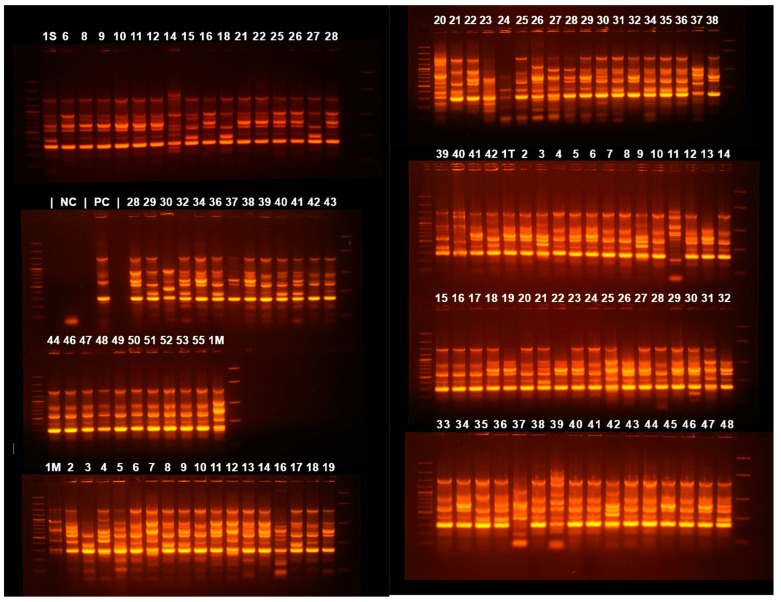
Agarose gel electrophoresis of ERIC-PCR amplification products. 1S—55 are samples from the Cluj-Napoca Infectious Diseases Clinical Hospital; 1M—55 from Cluj-Napoca Emergency County Hospital; 1T—42 from Târgu-Mureș Emergency County Hospital.

A dendrogram (Figure 3) was assembled based on ERIC-PCR electrophoresis patterns (Figure 4) of isolates obtained from all three hospitals. Mean dissimilarity was computed for each isolate group, and the total study population and clusters were designated following the establishment of a 0.1 threshold limit (90% similarity) as a reference point. The analysis showed that isolates from the Infectious Diseases Clinical Hospital had the highest degree of similarity (79.7%), isolates grouping into two main clusters under the 10% dissimilarity threshold. In comparison, the lowest degree of similarity belongs to the Cluj-Napoca Emergency County Hospital isolate group (57.35%) with five different clusters being established in order to encompass all isolates from this hospital. The total study population had a similarity percentage of 65.01%, being grouped into two main clusters, similar to the Infectious Diseases Clinical Hospital isolate group.

## 4. Discussion

The importance of *A. baumannii* as a causative agent of medical assistance-associated infections has been in the spotlight of microbiological and epidemiological research ever since its discovery in the late 1980s, gaining the top spot since its introduction into the ESKAPE list in 2017 by the WHO [1]. Although this species’ XDR CRAB phenotype represents a global concern nowadays, its presence in Southern and Eastern Europe is especially concerning due to its frequency of >50% of all reported isolates as of 2023 [12,43]. For a better understanding of the environmental resilience and spread of this phenotype, a characterization of its underlying genotypic profile and molecular fingerprinting studies are needed. To the best of our knowledge and database searches (PubMed, Web of Science), this is the first study on clinical isolates from confirmed infections obtained from different hospital settings in multiple cities across Romania.

Universal resistance to carbapenems (imipenem and meropenem), ciprofloxacin, and trimethoprim-sulfamethoxazole in XDR isolates confirms the limited efficacy of these antibiotics in treating various types of *A. baumannii* infections. In our study, the overwhelming majority of isolates (91.52%) were XDR, emphasizing the widespread presence of highly resistant bacteria in clinical environments. A study conducted in Serbia, a neighboring country in Eastern Europe, showed similar results in the predominant types of infection caused (respiratory infections, sepsis, and wound infections) and in the XDR CRAB phenotype isolation frequency (222 out of 237 isolates, 93.7%) [33]. Studies on isolates from Greece, Poland, and Slovakia report similarly high XDR phenotype isolation frequencies as well [44,45,46].

International reports show a variable but relatively less frequent presence of this phenotype [47,48,49], finding confirmed by a study on regional differences [50]. Recent studies from African countries provide updated data on the prevalence and resistance phenotypes of *A. baumannii*, with a meta-analysis covering data from sub-Saharan Africa between 2012 and 2022 finding a pooled prevalence of carbapenem-resistant *A. baumannii* (CRAB) at 8% (95% CI: 2–17%). Although this prevalence is relatively low compared to other regions, it underscores the impact CRAB phenotype has on African healthcare settings, particularly in intensive care units (ICUs) during this period [51]. A more recent study from Egypt in 2023 highlights a significant shift in resistance patterns. Analyzing 36 clinical isolates of *A. baumannii*, researchers reported markedly higher resistance rates compared to earlier studies (>90% of strains resistant to β-lactams (including carbapenems), 85–95% of strains resistant to fluoroquinolones and aminoglycosides, and 80% of strains resistant to trimethoprim-sulfamethoxazole) [52]. A comprehensive meta-analysis of *A. baumannii* isolates from six Asian countries, including China, India, Iran, Japan, South Korea, and Thailand, revealed high carbapenem resistance rates, with a pooled prevalence of resistance of 76.1% for imipenem, 73.5% for meropenem, and 74.3% for carbapenems overall. Resistance to meropenem peaked at 80.7% during the period from 2020 to 2023, indicating an upward trend in carbapenem resistance across the region [53]. In China, a study conducted in an intensive care unit (ICU) in Hangzhou over a three-month period revealed that 80.9% of A. baumannii isolates were carbapenem resistant (CRAB), with a newly emerging strain, ST164, accounting for 40.2% of the samples. This strain exhibited higher levels of antibiotic resistance compared to previously dominant strains, reflecting a significant shift in the resistance phenotype landscape [54]. In Latin America, the situation is equally concerning. A study conducted across six public hospital centers in Peru found that 85% of isolates were CRAB phenotypes, with a significant proportion harboring OXA-type carbapenemase genes, such as bla*_OXA-23_* and bla*_OXA-24/40_* [55].

The high frequency of an oxacillinase-encoding *bla_OXA-23__-like_* gene (91.5% of isolates) and *bla_OXA-24/40__-like_* gene (74.6% of isolates) highlights their involvement in the resistance phenotype. Alongside them, the *bla_NDM_* gene (25% of isolates) and *bla_IMP_* gene (0.84% of isolates) contribute to the carbapenem-resistant phenotype. In contrast, the *bla_OXA-51-like_* gene, a chromosomally encoded species marker, was only identified in 89% of isolates in spite of the very accurate identification using MALDI-TOF-MS and Vitek 2. This finding possibly reflects mutations in the primer binding sites or the involvement of IS*Aba1* or another insertion sequence being present upstream of the *bla_OXA-51-like_* gene, which could alter the sequence near the primer binding sites, thus preventing the primers from annealing effectively [56]. However, technical PCR limitations cannot be entirely ruled out. This finding was similar to another study that found 87.6% of isolates positive for this gene [57]. We have also identified the insertion sequence IS*Aba1* in 104 isolates (88%), and its upstream placement relative to the *bla_OXA-23__-like_* and *bla_OXA-51-like_* genes was observed in 80 isolates (68%). When IS*Aba1* is located upstream of an oxacillinase-encoding gene, it can lead to higher expression levels of the oxacillinase enzyme, enhancing resistance to carbapenems like imipenem or meropenem [31,44,56,58]. Overexpressed oxacillinases (due to IS*Aba1*) work synergistically with MBLs, leading to higher-level resistance in comparison with the effect of either enzyme alone. This combination, along with *A. baumannii*-derived cephalosporinase (an Ambler C class chromosomally encoded beta-lactamase), makes infections very difficult to treat, as these enzymes target different categories of beta-lactam antibiotics, leaving few effective therapeutic options [59]. One possible explanation for the presence of IS*Aba1* upstream of the *bla_OXA-23_* gene is that the isolates may have acquired it by means of one of the four transposons containing this sequence [60].

Resistance to all three tested aminoglycosides was found in 88.88% of XDR isolates, with 11.11% retaining partial sensitivity, which might provide limited therapeutic options in combination regimens. The large proportion of *ArmA* (63.6%) and *ant(3”)-I* (58.5%) genes, encoding a 16S rRNA methyltransferase and an aminoglycoside-modifying enzyme, respectively, reinforces their role in mediating high-level aminoglycoside resistance. Less prevalent genes such as *aac(6′)-Ib* (15.3%) and *aac(3′)-II* (6.8%) also contribute to the resistance landscape to a lesser extent. In total, all isolates displaying one of the aforementioned genes were resistant to at least one aminoglycoside. By contrast, there were 11 XDR isolates (9.32%) with resistance to two or all three aminoglycosides and none of the tested AME or 16S rRNA methyltransferase genes present, implying that there are other possible mechanisms responsible for aminoglycoside resistance, such as efflux pumps and modification of membrane permeability [61].

Quinolone resistance was identified in all MDR and XDR isolates, with only nine isolates (7.62%) testing positive for *qnrS* protein protecting DNA gyrase and topoisomerase IV from the effect of quinolones. This finding suggests that the main driver of quinolone resistance in *A. baumannii* hospital isolates lies in other mechanisms, point mutations in *gyrA* and *parC* genes encoding DNA gyrase, and topoisomerase IV having the highest impact alongside efflux pumps [62].

For trimethoprim-sulfamethoxazole resistance, the focus is on acquiring mechanisms generating sulfonamide resistance since *A. baumannii* is intrinsically resistant to trimethoprim due to its lack of effect on this species’ dihydrofolate reductase (DHFR) enzyme. The main mechanism of resistance is the production of modified dihydropteroate synthase (DHPS) enzymes that cannot be inhibited by sulfonamides, production mediated by *sul1, sul2*, and *sul3* genes. This process is confirmed by our study findings, *sul1* genes being present in 113 isolates (95.76%). However, we also discovered three resistant isolates (2.54%) with no *sul1* gene, where resistance to sulfonamides may be due to other structures [63].

Colistin was the primary antibiotic used to treat *A. baumannii* infections, administered as monotherapy in 48.3% of cases and in combination regimens, most commonly with meropenem (17.79%) or tigecycline (6.77%). Sensitivity to colistin remains generally intact, consistent with its status as a last-resort treatment, though concerns about emerging colistin resistance remain (3.38% of the isolates in these studies were PDR). For the colistin-resistant isolates, we tested for the acquisition of *mcr-1* gene encoding a phosphoethanolamine transferase, which modifies the lipid A in the outer membrane and *mexA* and *mexB* genes encoding proteins of the *MexA*B-OprM efflux pump, with none of the isolates testing positive. The literature describes the aforementioned mechanisms of colistin resistance predominantly in *Escherichia coli* and *Pseudomonas aeruginosa,* but they can be present in *A. baumannii* through HGT [64]. The continued reliance on colistin, despite its well-documented nephrotoxicity and neurotoxicity [65,66,67], underscores its critical role in managing extensively drug-resistant infections. Combination therapies are often employed to enhance its efficacy and mitigate resistance. These findings highlight the limited treatment options available and the significant challenges in managing *A. baumannii* infections.

The in-depth association between resistance phenotypes and resistance genotype patterns could have multiple implications for diagnosis and future antibiotic usage optimization. Screening for IS*Aba1* upstream of resistance genes, especially OXA-type carbapenemase genes, could predict upregulated resistance phenotypes, supporting early and targeted therapeutic interventions. Screening for molecular markers that can be integrated into diagnostic tools will enable a rapid detection of resistant strains, even before conventional culture and susceptibility tests are completed. Furthermore, understanding the resistome highlights potential targets for new antimicrobials or adjuvants. For example, inhibitors of efflux pumps or enzymes encoded by genes like *bla_OXA-23-like_* could restore the efficacy of existing antibiotics.

A high similarity between isolates might be indicative of a cluster of carbapenem-resistant *A. baumannii* involved in a hospital outbreak rather than being community acquired since this species has been previously implicated in such outbreaks [68]. Local transmission of *Acinetobacter* spp. is facilitated by its persistence on abiotic surfaces and colonization of the hands of healthcare workers [69,70]. We found differences in isolate similarity between hospitals, suggesting a variability in transmission. However, the lack of statistical association between resistance phenotypes and hospitals, wards, or infection types suggests a widespread dissemination of resistant isolates rather than localized outbreaks.

Differences in infection prevention and control (IPC) measures can greatly impact the clonal similarity of bacteria between hospitals. This hypothesis is confirmed by studies regarding the correlation between IPCs and the frequency of multi-drug-resistant species isolation from clinical settings. Fewer IPC measures were generally implemented by hospitals with the highest prevalence of multi-drug-resistant species. Those who have the lowest multi-drug-resistant species prevalence adopt more IPC measures. They also better adhere to their policies. Multi-drug-resistance endemicity or resource limitations may cause differences in IPC strategies. Increased adherence to IPC policies could lead to a large reduction in the prevalence of such isolates, thereby benefiting overall public health and safety [71,72,73].

## 5. Conclusions

*A. baumannii* is a notoriously efficient species when it comes to survivability in hospital settings and medical assistance-associated infections. This is in part due to the high prevalence of XDR isolates with limited therapeutic options to counter them, with colistin often being the only viable antibiotic. However, reliance on colistin heightens the risk of resistance development through selective pressure, emphasizing the urgent need for novel antimicrobial agents and combination therapies. The widespread distribution of XDR isolates across hospitals and infection types calls for enhanced infection prevention measures, particularly in high-risk areas such as ICUs. Last but not least, the genetic diversity of resistance mechanisms and acquisition methods highlights the importance of continuous molecular surveillance in addition to the phenotype monitorization in order to precisely track resistance trends and devise new and improved infection control policies.

## Figures and Tables

**Table 1:** PCR primer gene names, sequences, annealing temperatures, and amplicon sizes for the detected antibiotic resistance genes and *MexA*B efflux pumps.

Gene Name	Primer Sequence (5′-3′)	Annealing t °C	Amplicon Size	Reference
*bla_OXA-23-like_*	Fw-GATCGGATTGGAGAACCAGA	58 °C	501 bp	[31]
	Rev-ATTTCTGACCGCATTTCCAT			
*bla_OXA-24/40-like_*	Fw-GGTTAGTTGGCCCCCTTAAA	59 °C	246 bp	[31]
	Rev-AGTTGAGCGAAAAGGGGATT			
*bla_OXA-51-like_*	Fw-TAATGCTTTGATCGGCCTTG	58 °C	353 bp	[31]
	Rev-TGGATTGCACTTCATCTTGG			
*bla_OXA-143-like_*	Fw-TGGCACTTTCAGCAGTTCCT	61 °C	149 bp	[32]
	Rev-TAATCTTGAGGGGGCCAACC			
*bla_NDM_*	Fw-GGTTTGGCGATCTGGTTTTC	61 °C	621 bp	[33]
	Rev-CGGAATGGCTCATCACGATC			
*bla_VIM_*	Fw-ATTGGTCTATTTGACCGCGTC	58 °C	780 bp	[34]
	Rev-TGCTACTCAACGACTGAGCG			
*bla_IMP_*	Fw-GGAATAGAGTGGCTTAATTC	52 °C	232 bp	[31]
	Rev-TCGGTTTAATAAAACAACCACC			
*bla_SIM_*	Fw-TACAAGGGATTCGGCATCG	59 °C	570 bp	[35]
	Rev-TAATGGCCTGTTCCCATGTG			
*bla_SPM_*	Fw-AAAATCTGGGTACGCAAACG	57 °C	271 bp	[36]
	Rev-ACATTATCCGCTGGAACAGG			
*aac(6′)-Ib*	Fw-CATGACCTTGCGATGCTCTA	62 °C	490 bp	[37]
	Rev – GCTCGAATGCCTGGCGTCTT			
*aac(3′)-IIc*	Fw-ACGCGGAAGGCAATAACGGA	61 °C	854 bp	[37]
	Rev-TAACCTGAAGGCTCGCAAGA			
*ant(3”)-I*	Fw-TGATTTGCTGGTTACGGTGAC	46 °C	284 bp	[37]
	Rev-CGCTATGTTCTCTTGCTTTTG			
*aph(3′)-IIb*	Fw-ATGCATGATGCAGCCACCTCC	51 °C	804 bp	[37]
	Rev-CTAGAAGAACTCGTCCAATAGCCT			
*ArmA*	Fw-TGGGAAGTTAAAGACGACGA	56 °C	315 bp	[37]
	Rev-CCATTCCCTTCTCCTTTCCA			
*sul1*	Fw-AGGCATGATCTAACCCTCGG	62 °C	665 bp	[38]
	Rev-GGCCGATGAGATCAGACGTA			
*qnrA*	Fw-AGTTTGATGGTTGCCGCTTT	59 °C	541 bp	[38]
	Rev-TCTTCATTGATCTGCACGCC			
*qnrB*	Fw-TCGTGCGATGCTGAAAGATG	60 °C	368 bp	[38]
	Rev-CCGAATTGGTCAGATCGCAA			
*qnrS*	Fw-TGATCTCACCTTCACCGCTT	60 °C	496 bp	[38]
	Rev-GAGTTCGGCGTGGCATAAAT			
IS*Aba1*	Fw-CACGAATGCAGAAGTTG	51 °C	550 bp	[39]
	Rev-CGACGAATACTATGACAC			
IS*Aba1/bla_OXA-23-like_*	Fw- AATGATTGGTGACAATGAAG	55 °C	1433 bp	[40]
	Rev- ATTTCTGACCGCATTTCCAT			
IS*Aba1/bla_OXA-51-like_*	Fw- AATGATTGGTGACAATGAAG	56 °C	1252 bp	[40]
	Rev- TGGATTGCACTTCATCTTGG			
*MexA*	Fw-ATCAACCTGCGCTACACCAAG	61 °C	291 bp	[41]
	Rev-AGGCCTTCGGTAATGATCTTGT			
*MexB*	Fw-TTTCATTGATAGGCCCATTTTC	57 °C	353 bp	[41]
	Rev-AGGGTCTTCACTACCTCATGGA			
*mcr-1*	Fw-CGGTCAGTCCGTTTGTTC	58 °C	320 bp	[42]
	Rev-CTTGGTCGGTCTGTAGGG			

**Table 2:** PCR primer gene names, sequences, annealing temperatures, and amplicon sizes for integrase genes amplifications.

Integron Class	Targeted Gene	Primer Sequence (5′-3′)	Annealing t °C	Amplicon Size	Reference
Class I	*int1*	Fw-CAGTGGACATAAGCCTGTTC	58 °C	160 bp	[38]
		Rev-CCCGACGCATAGACTGTA			
Class II	*int2*	Fw-TTGCGAGTATCCATAACCTG		788 bp	[38]
		Rev-TTACCTGCACTGGATTAAGC			

**Table 3:** PCR primer gene names and sequences for ERIC-PCR amplification.

ERIC-PCR Primer	Primer Sequence (5′-3′)	Reference
ERIC1R	TGTAAGCTCCTGGGGATTCAC	[30]
ERIC2	AAGTAAGTGACTGGGGTGAGCG	

**Table 4:** General and clinical data regarding inpatients with *A. baumannii* infections.

Data	Nr. and % of Inpatients
Sex	
Male	70 (59.32%)
Female	48 (40.67%)
Age group (years old)	
<18	4 (3.38%)
18–30	3 (2.54%)
31–45	5 (4.23%)
46–60	24 (20.33%)
61–75	45 (38.13%)
>76	37 (31.35%)
Admission ward	
ICU	50 (42.37%)
Medical wards	20 (16.94%)
General surgery	17 (14.4%)
Neurology	10 (8.47%)
Neurosurgery	8 (6.77%)
Orthopedics	3 (2.54%)
Nephrology	3 (2.54%)
Cardiology	2 (1.69%)
Cardiovascular surgery	2 (1.69%)
Obstetrics–Gynecology	1 (0.84%)
Immunosuppressed patients ward	1 (0.84%)
Neonatology	1 (0.84%)
Type of infection	
Respiratory tract infections	54 (45.76%)
Sepsis	32 (27.11%)
Wound infections	20 (16.94%)
Urinary tract infections	6 (5.08%)
Abscess	3 (2.54%)
Peritonitis	2 (1.69%)
Meningitis	1 (0.84%)
Collected specimen	
Tracheal aspirate	52 (44.06%)
Blood	22 (18.64%)
Wound exudate	22 (18.64%)
Urine	6 (5.08%)
Bronchoalveolar lavage fluid	5 (4.23%)
Sputum	4 (3.38%)
Pus	2 (1.69%)
Central venous catheter tip	2 (1.69%)
Peritoneal fluid	2 (1.69%)
Cerebrospinal fluid	1 (0.84%)
Outcome	
Deceased	64 (54.23%)
Worsened	1 (0.84%)
Stationary	13 (11.01%)
Improved	34 (28.81%)
Cured	6 (5.08%)

**Table 5:** A contingency table of the frequency of beta-lactamase-encoding ARGs and resistance phenotype.

	Negative/Positive (0/1)	Meropenem		Imipenem		*p*-Value Meropenem	*p*-Value Imipenem
		**S**	**R**	**S**	**R**		
*bla_OXA-23-like_*	0	2	8	2	8	NS	NS
	1	3	105	3	105		
*bla_OXA-24/40-like_*	0	1	29	1	29	NS	NS
	1	4	84	4	84		
*bla_OXA-51-like_*	0	3	10	3	10	*p* < 0.01	*p* < 0.01
	1	2	103	2	103		
IS*Aba1*	0	2	12	2	12	NS	NS
	1	3	101	3	101		
IS*Aba1*/*bla_OXA-23-like_*	0	5	33	5	33	*p* < 0.01	*p* < 0.01
	1	0	80	0	80		
IS*Aba1*/*bla_OXA-51-like_*	0	5	33	5	33	*p* < 0.01	*p* < 0.01
	1	0	80	0	80		
*bla_NDM_*	0	5	84	5	84	NS	NS
	1	0	29	0	29		
*bla_IMP_*	0	5	112	5	112	NS	NS
	1	0	1	0	1		

**Table 6:** Contingency table of aminoglycoside ARGs and resistance phenotype frequency.

	Negative/Positive (0/1)	Amikacin		Gentamycin		Tobramycin		*p*-Value Amikacin	*p*-Value Gentamycin	*p*-Value Tobramycin
		**S**	**R**	**S**	**R**	**S**	**R**			
*aac(6′)-Ib *	0	10	90	8	92	12	88	NS	NS	NS
	1	0	18	1	17	1	17			
*aac(3′)-II *	0	9	101	9	101	12	98	NS	NS	NS
	1	1	7	0	8	1	7			
*ant(3”)-I *	0	7	42	5	44	8	41	NS	NS	NS
	1	3	66	4	65	5	64			
*ArmA *	0	8	35	8	35	9	34	*p* < 0.01	*p* < 0.01	*p* < 0.05
	1	2	73	1	74	4	71			

## Data Availability

Information about antibiotic resistance genes were curated from The Comprehensive Antibiotic Resistance Database (https://card.mcmaster.ca, accessed on 3 December 2024). All data generated or analyzed during this study are included in this published article.
